# Levetiracetam-Induced Rhabdomyolysis: An Alarming and Unexpected Turn in Seizure Management

**DOI:** 10.7759/cureus.91435

**Published:** 2025-09-01

**Authors:** Usamah Al-Anbagi, Ahmad E Alharafsheh, Bassem Al Hariri, Memon Noor Illahi, Abdulqadir J Nashwan, Muhammad Sharif

**Affiliations:** 1 Internal Medicine Department, Hazm Mebaireek General Hospital/Hamad Medical Corporation, Doha, QAT; 2 Pharmacy Department, Hamad Medical Corporation, Doha, QAT; 3 Internal Medicine Department, Hamad Medical Corporation, Doha, QAT; 4 Nursing and Midwifery Research Department, Hamad Medical Corporation, Doha, QAT

**Keywords:** case report, creatine kinase (ck), drug induced rhabdomyolysis, levetiracetam, rhabdomyolysis, seizure

## Abstract

Levetiracetam is a widely used antiepileptic drug favored for its efficacy and favorable safety profile. However, rare cases of levetiracetam-induced rhabdomyolysis have been reported, posing diagnostic and therapeutic challenges. We describe a 29-year-old male with a psychiatric history who presented with a generalized tonic-clonic seizure. The patient was started on levetiracetam for seizure management. Despite initial clinical improvement, creatine kinase levels rose dramatically, exceeding 22,000 U/L, consistent with severe rhabdomyolysis. Levetiracetam was discontinued and replaced with valproic acid, resulting in significant creatine kinase reduction and clinical recovery. This case highlights the importance of recognizing levetiracetam as a potential, albeit rare, cause of rhabdomyolysis. Early identification and prompt discontinuation of the drug, combined with supportive care, are crucial to prevent serious complications such as acute kidney injury. Clinicians should maintain vigilance when monitoring patients on levetiracetam, especially if creatine kinase levels rise unexpectedly.

## Introduction

Levetiracetam has become a cornerstone in the management of various seizure disorders due to its broad-spectrum efficacy, favorable pharmacokinetics, and minimal drug interactions [1]. Its mechanism involves binding to synaptic vesicle protein 2A (SV2A), modulating neurotransmitter release, and stabilizing neuronal activity [2]. While generally well tolerated, levetiracetam has still been associated with some reported adverse effects. Commonly reported side effects include behavioral changes, fatigue, and dizziness; however, severe muscular complications such as rhabdomyolysis remain exceedingly rare and underrecognized [2].

Rhabdomyolysis, characterized by the breakdown of skeletal muscle and the release of intracellular components into the circulation, can lead to acute kidney injury and electrolyte imbalances, posing significant risks to morbidity and mortality [3]. It is often precipitated by trauma, seizures, medications, or metabolic disturbances [4]. Although seizures themselves can elevate creatine kinase (CK) levels, persistent or severe elevations warrant further investigation for underlying causes, including drug-induced myopathy [5]. This report presents a rare case of severe rhabdomyolysis associated with levetiracetam, underscoring the need for awareness and timely management of this potentially life-threatening adverse effect.

## Case presentation

A 29-year-old man with a known psychiatric illness was recently discharged from a psychiatric hospital, where he had been admitted for delusional thoughts (not known to have any seizure disorder). At discharge, he was prescribed sertraline and clozapine (the dose of sertraline was 50 mg oral daily, and the clozapine was 0.5 mg oral once a day). He was brought to the emergency department by ambulance after a witnessed generalized tonic-clonic seizure at home, as reported by his friends. Upon arrival, he was drowsy and confused, with no ongoing seizure activity (The event was witnessed toward its end; therefore, the exact duration is unknown. This was the first episode, as the patient had no prior history of similar events).

On general examination, vital signs were as follows: T: 37 °C, HR: 63/min, RR: 18/min, BP: 134/83 mmHg, SpO₂: 98% on room air. There was no pallor, jaundice, cyanosis, clubbing, or thyromegaly. Head and neck examination revealed evidence of right frontal trauma post-seizure. The chest examination revealed normal vesicular breath sounds bilaterally on room air, with no additional sounds. Cardiovascular examination revealed normal heart sounds (S1 and S2), no audible murmurs, and intact peripheral pulses. The abdomen was soft, non-tender, non-distended, with no organomegaly or free fluid. The neurological examination revealed equal and reactive pupils. The patient was spontaneously moving all four limbs but was drowsy and uncooperative. There was no tremor or abnormal movement, and muscle power was 5/5 in all four limbs; the tone was normal in all four limbs. Spine examination was normal. 

**Figure 1:**
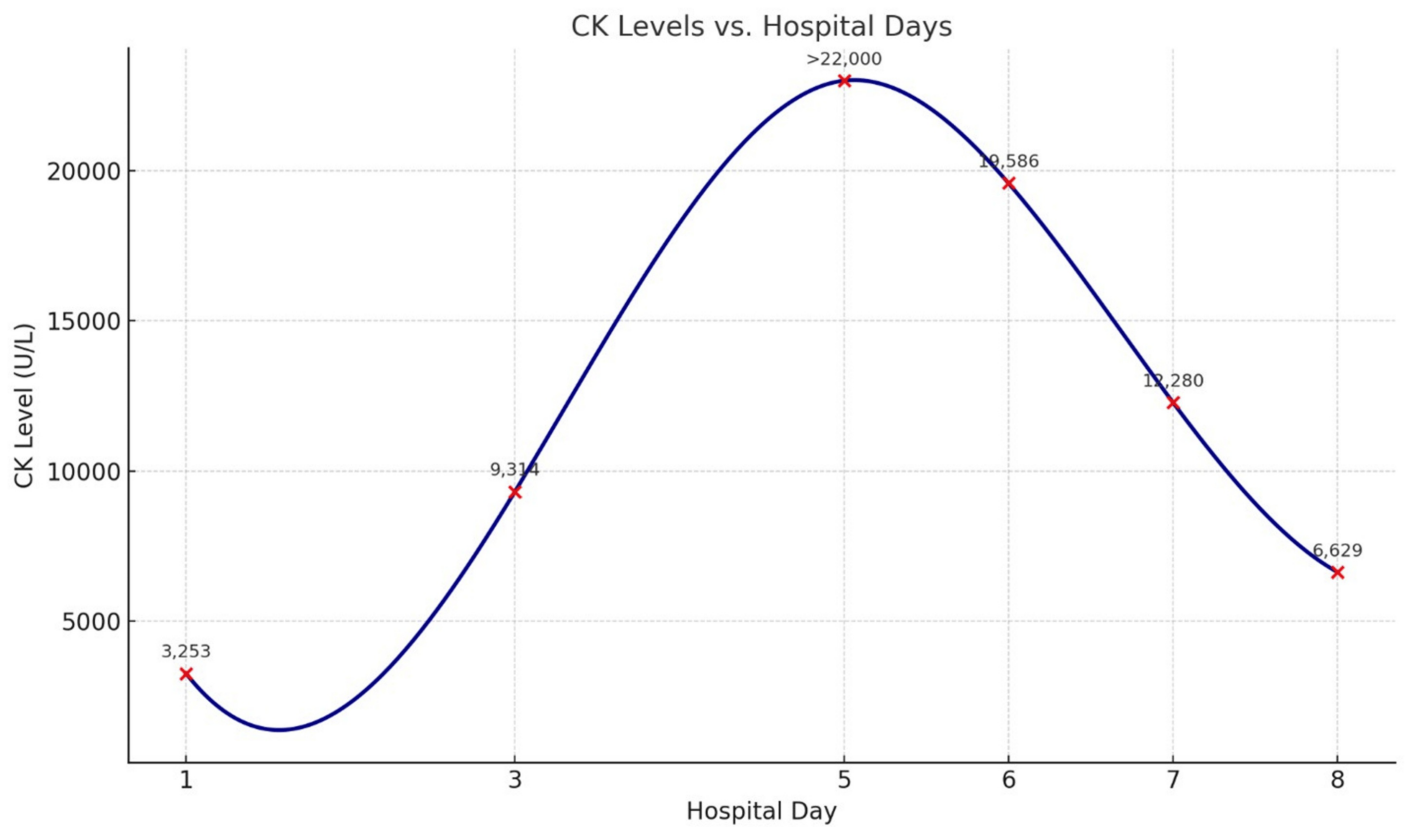
The trend of creatine kinase levels over the course of hospitalization

He was admitted with a case of seizure in the post-ictal phase. Initial laboratory investigations revealed severe hyponatremia, moderate hypomagnesemia, and mild hypokalemia; the rest of the routine tests were within acceptable limits (Table 1). The hyponatremia was considered secondary to sertraline induced SSRI, as no other obvious cause was identified. He was started on levetiracetam 500 mg IV twice daily, along with a single dose of lorazepam 2 mg. CK was checked at the end of the first day of admission and was elevated at 3,253 U/L, which was initially attributed to seizure-related muscle activity. By the second day, with sodium correction and levetiracetam treatment, the patient showed progressive clinical improvement and returned to his baseline status. He was evaluated by a neurologist, who recommended continuing levetiracetam. The IV formulation was continued for three days before switching to oral levetiracetam 500 mg BID. A brain MRI was performed and was unremarkable. On Day 3, repeat CK levels showed a significant rise to 9,314 U/L, and two days later, results exceeded the measurable range (>22,000 U/L) (Figure 1), prompting concern for levetiracetam-induced rhabdomyolysis. After discussion with the neurology team, levetiracetam was discontinued and replaced with valproic acid 500 mg BID, along with vigorous IV fluid hydration.

**Table 1:** Laboratory investigations ALT: alanine aminotransferase; AST: aspartate aminotransferase; TSH: thyroid-stimulating hormone; FT4: free thyroxine; PT: prothrombin time; INR: international normalized ratio; APTT: activated partial thromboplastin time; CK: creatine kinase; pH: potential of hydrogen

Parameters	On admission	On Day 3	On Day 5	On discharge	Reference values
Serum urea (mmol/L)	2.2	3.3	4.5	4.2	2.5-7.8
Serum creatinine (umol/L)	78	68	79	91	62-106
Serum potassium K (mmol/L)	3.4	4.2	4.6	4.9	3.5-5.3
Serum sodium (mmol/L)	116	142	138	139	133-146
Serum phosphorus (mmol/L)	1.02	1.2	-	-	0.8-1.5
Serum magnesium (mmol/L)	0.56	0.74	-	0.77	0.7-1 mmol/L
PH	7.4	-	-	-	7.320-7.420
Serum total protein (gm/L)	63	-	-	-	60-80
Serum albumin (gm/L)	39	-	-	-	35-50
ALT (IU/L)	13	-	-	-	0-41
AST (IU/L)	29	-	-	-	0-41
Alkaline phosphatase (U/L)	59	-	-	-	40–129
Serum total bilirubin (mg/dl)	11	-	-	-	0-21
TSH (mIU/L)	1.92	-	-	-	0.34-4.20 mIU/L
FT4 (pmol/L)	17.9	-	-	-	11-23.3 pmol/L
PT (seconds)	12.6	-	-	-	9.4-12.5
INR	1.1	-	-	-	<1
APTT (seconds)	31.5	-	-	-	25.1- 36.5
Creatinine kinase (U/L)	3253	9314	>22000	6,629	39-3.8 U/L

CK levels subsequently began to decline significantly, reaching 6,620 U/L three days later (Table 1). The patient was discharged in good clinical condition with the final diagnosis of seizure secondary to hyponatremia and severe rhabdomyolysis induced by levetiracetam. He was discharged on valproic acid 500 mg BID, with follow-up arranged in neurology and psychiatry clinics.

## Discussion

In this case, we describe a previously healthy young man with a psychiatric history who developed severe rhabdomyolysis shortly after the initiation of levetiracetam for a new-onset seizure. While an initial mild elevation in CK was attributed to seizure-related muscle injury, the persistent and dramatic rise in CK levels over subsequent days, in the absence of ongoing seizures or other risk factors, raised suspicion for a drug-induced cause. The temporal association with levetiracetam initiation, along with the significant improvement following its discontinuation and supportive treatment, strongly supports a causal relationship. This case draws attention to a rare but serious adverse effect of a commonly used antiepileptic medication.

Rhabdomyolysis is a clinical syndrome caused by the breakdown of skeletal muscle tissue, resulting in the release of intracellular enzymes and electrolytes into the bloodstream. Common causes include trauma, prolonged immobilization, seizures, electrolyte imbalances, and a range of medications. Among the drug-related causes, statins, certain antipsychotics, and older-generation antiepileptics such as phenytoin and valproic acid are well-documented offenders [3,5]. The severity of rhabdomyolysis can vary widely, from asymptomatic CK elevation to life-threatening complications such as acute kidney injury and electrolyte disturbances. Seizures themselves can also cause transient CK elevation, which can complicate the interpretation of laboratory results in post-ictal patients [3,5].

Levetiracetam is a widely used antiepileptic agent known for its favorable safety profile, lack of hepatic metabolism, and minimal drug-drug interactions. Despite this, a growing number of case reports have described an association between levetiracetam and the development of rhabdomyolysis, even in otherwise healthy individuals without other identifiable risk factors [6-8]. In most of these reports, CK levels rose significantly within a few days of drug initiation and declined following its discontinuation, suggesting a probable causal relationship. The exact incidence remains unclear due to the rarity of reported cases, but it may be underrecognized, especially when CK elevation is misattributed to seizure activity alone [6-8]. These cases, including ours, underscore the importance of considering levetiracetam as a potential cause of rhabdomyolysis in patients with unexplained or worsening CK elevations.

The precise mechanism by which levetiracetam induces rhabdomyolysis remains unclear, as this adverse effect is exceedingly rare and not fully understood. Generally, rhabdomyolysis can result from direct muscle injury, metabolic disturbances, or drug-induced muscle toxicity, often involving mitochondrial dysfunction or disruption of calcium homeostasis within muscle cells [3,5]. Although levetiracetam is not traditionally associated with muscle toxicity, some hypotheses suggest it may trigger muscle damage through idiosyncratic immune-mediated reactions or by interfering with muscle cell metabolism in susceptible individuals [8,9]. Additionally, levetiracetam’s modulation of SV2A, while beneficial in controlling seizures, could potentially have off-target effects on muscle tissue that are not yet fully characterized [10]. Further research is needed to elucidate the exact pathophysiological processes involved, but awareness of this potential mechanism is crucial for early recognition and prevention of severe muscle injury.

Prompt recognition and discontinuation of the offending agent are critical in managing drug-induced rhabdomyolysis. In this case, stopping levetiracetam and initiating intravenous hydration led to a marked reduction in CK levels and prevented further complications such as acute kidney injury. Supportive care remains the cornerstone of treatment, focusing on maintaining renal perfusion and correcting electrolyte imbalances [3,5]. The switch to valproic acid provided continued seizure control without recurrence of muscle injury, highlighting the importance of individualized therapy when adverse drug reactions arise. Close monitoring during the transition and follow-up ensured favorable clinical outcomes and minimized potential sequelae [11].

This case underscores the necessity for clinicians to maintain a high index of suspicion for rhabdomyolysis in patients on levetiracetam, especially when CK levels rise unexpectedly or clinical symptoms suggest muscle injury. Although rare, levetiracetam-induced rhabdomyolysis can be severe and life-threatening if not promptly identified and managed [2]. Routine monitoring of CK may be warranted in select patients, particularly those presenting with myalgias, weakness, or unexplained laboratory abnormalities. Moreover, this case emphasizes the value of multidisciplinary collaboration to optimize patient care and tailor antiepileptic regimens safely [4]. Increased awareness can lead to early detection, prevent serious complications, and improve patient outcomes.

## Conclusions

Levetiracetam-induced rhabdomyolysis, although rare, represents a serious adverse effect that clinicians should keep in mind when managing patients on this medication. This case underscores the importance of monitoring muscle enzymes and recognizing the early signs of muscle injury to prevent potentially life-threatening outcomes. Timely cessation of levetiracetam and initiation of supportive measures can lead to rapid recovery and prevent complications. Awareness of this uncommon but significant risk enables safer prescribing practices and enhanced patient care.
